# Nutrient Patterns, Cognitive Function, and Decline in Older Persons: Results from the Three-City and NuAge Studies

**DOI:** 10.3390/nu11081808

**Published:** 2019-08-05

**Authors:** Benjamin Allès, Cécilia Samieri, Marthe-Aline Jutand, Pierre-Hugues Carmichael, Bryna Shatenstein, Pierrette Gaudreau, Guylaine Ferland, Pascale Barberger-Gateau, Danielle Laurin

**Affiliations:** 1 University Bordeaux, Inserm, Bordeaux Population Health Research Center, UMR 1219, F-33000 Bordeaux, France; 2 Faculté de Pharmacie, Laval University, Québec, QC G1V 0A6, Canada; 3 Centre d’excellence sur le vieillissement de Québec, Centre de recherche du CHU de Québec, Institut sur le vieillissement et la participation sociale des aînés (IVPSA), Québec, QC G1S 4L8, Canada; 4 Centre de recherche sur les soins et les services de première ligne de l’Université Laval (CERSSPL-UL), Québec, QC G1J 0A4, Canada; 5 Equipe de Recherche en Epidémiologie Nutritionnelle, Centre de Recherche en Epidémiologie et Statistiques, Université Paris 13, Inserm (U1153), Inra (U1125), Cnam, Université de Paris, 93017 Bobigny, France; 6 Culture et Diffusion des Savoirs EA-7440, University Bordeaux, F-33000 Bordeaux, France; 7 Département de Nutrition, Université de Montréal, Québec and Centre de recherche, Institut universitaire de gériatrie de Montréal, QC H3T 1A8, Canada; 8 Department of Medicine, University of Montreal and Centre Hospitalier de l’Université de Montréal Research Center (CRCHUM), Montréal, QC H3T 1J4, Canada; 9 Canadian Consortium on Neurodegeneration in Aging-Nutrition, Exercise and Lifestyle Team, Montréal, H3T 1E2, Canada; 10 Département de Nutrition, Université de Montréal, Québec and Research Centre, Montreal Heart Institute, Montréal, QC H1T 1C8, Canada

**Keywords:** diet, cognition, aging, nutrition, nutrients, principal component analysis

## Abstract

Dietary patterns, or the combination of foods and beverages intake, have been associated with better cognitive function in older persons. To date, no study has investigated the link between a posteriori nutrient patterns based on food intake, and cognitive decline in longitudinal analyses. The aim of this study was to evaluate the relationship between nutrient patterns and cognitive function and decline in two longitudinal cohorts of older persons from France and Canada. The study sample was composed of participants from the Three-City study (3C, France) and the Quebec Longitudinal Study on Nutrition and Successful Aging (NuAge, Quebec, Canada). Both studies estimated nutritional intakes at baseline, and carried out repeated measures of global cognitive function for 1,388 and 1,439 individuals, respectively. Nutrient patterns were determined using principal component analysis methodology in the two samples, and their relation with cognitive function and decline was estimated using linear mixed models. In 3C, a healthy nutrient pattern, characterized by higher intakes of plant-based foods, was associated with a higher global cognitive function at baseline, as opposed to a Western nutrient pattern, which was associated with lower cognitive performance. In NuAge, we also found a healthy nutrient pattern and a Western pattern, although no association was observed with either of these patterns in the Canadian cohort. No association between any of the nutrient patterns and cognitive decline was observed in either cohort. There is a need for longitudinal cohorts focusing on nutrient patterns with substantial follow-up, in order to evaluate more accurately associations between nutrition and cognition in older persons.

## 1. Introduction

In aging societies, concern for cognitive health is on the rise. In the absence of an effective treatment for Alzheimer’s disease (AD), identification of modifiable risk factors that could delay or prevent its symptomatic stage has become a public health research priority [1,2]. Indeed, the symptomatic stage of AD, including the loss of cognitive function, represents a burden not only for those who suffer this disease, but also for their caregivers [3]. Diet has been suggested to play a role in maintaining the integrity of cognitive function, but results from experimental and epidemiological studies based on a single nutrient approach are mixed [4]. The combination of a set of nutrients or food groups into patterns may better capture the complexity of food intake [5]. A recent literature review reported that the evidence of a beneficial effect on cognitive outcomes was more convincing for healthy dietary patterns than for single foods or nutrients [6].

Dietary and nutrient patterns can be computed in two ways [4,7]. A priori patterns are defined using diet indices that measure adherence to a specific diet, such as the Mediterranean diet, or to recommended dietary guidelines for a healthy diet [8]. Limitations of the a priori patterns are that they are hypothesis-driven and do not account for the total food intake, but only for some components of the diet. In contrast, a posteriori patterns summarize the whole dietary intake into a few representative profiles using exploratory statistical methods. In a recent systematic review on dietary patterns and cognitive health in older adults including 37 studies, only 6 studies used a posteriori dietary patterns [9]. In Australia, a prudent healthy a posteriori dietary pattern was not associated with cognition [10]. In contrast, in Sweden, higher adherence to a prudent a posteriori healthy dietary pattern was associated with a lower global cognitive decline, whereas a higher adherence to a Western dietary pattern was associated with a greater global cognitive decline [11]. Among the other selected studies using a posteriori dietary patterns included in the systematic review, a Western dietary pattern was consistently associated with lower cognitive health [9].

Very limited research has examined a posteriori nutrient patterns in relation to cognitive function [12]. Reflecting the global quality of dietary intake, nutrient patterns could be a more accurate means of comparison of dietary habits in different study settings—for example, in different cohorts of older adults [13]. We previously conducted such a comparative study in two older populations. This study allowed us to identify and describe nutrient patterns in the 3C study (France) and the Québec Longitudinal Study on Nutrition and Successful Aging (NuAge, Québec, Canada) [13]. Similar healthy and Western nutrient patterns were observed in both cohorts, whereas a third pattern appeared to reflect traditional cultural or geographical dietary habits specific to each population. The aim of the present study was to evaluate the relationship of these nutrient patterns to global cognitive function and decline in these two cohorts of older persons, over a period of up to five years.

## 2. Methods

### 2.1. Study Populations

*3C study, France.* The 3C study is an ongoing longitudinal study of vascular risk factors for dementia, which includes 9294 community dwellers aged 65 years and over, randomly selected from electoral rolls from Bordeaux (*n* = 2104), Dijon (*n* = 4931), and Montpellier (*n* = 2259) [14]. The baseline examination was carried out in 1999–2000 with face-to-face interviews; participants have been re-evaluated at home every two to three years. Data collection included socio-demographic and lifestyle characteristics, medical history, comprehensive neuropsychological testing, physical examination, and blood sampling. At the first follow-up (2001/2002), 1712 participants from the Bordeaux sample further answered an extensive dietary survey conducted by trained dietitians. The current study is based on data collected between 2001 and 2007.

*NuAge Study, Québec.* The NuAge study is a longitudinal study focusing on the role of nutrition as determinant of a successful aging. Participants were living in the Montreal, Laval, or Sherbrooke areas in Québec (Canada) and were selected randomly from the Québec administrative health database, after stratification for age and sex. The original sample included 1587 individuals recruited between November 2003 and June 2005 (T1), to which 206 volunteers were added [15]. They were community-dwelling men and women, aged 67–84 years and in good general health [15]. Notably, subjects should not be cognitively impaired based on a score of >79 on the Modified Mini-Mental State examination (3MS) [15,16]. The 3MS is a 100-point validated questionnaire assessing global cognitive function. NuAge participants were re-examined annually, and followed 3 times (T2, T3, T4) over a 4-year period. Nutritional data were collected by trained dietitians in structured face-to-face and telephone interviews [16]. From the original sample of 1793 NuAge participants, 1754 (98%) agreed to the integration of their data into the NuAge Database and Biobank for future studies. The current study is based on data collected between 2003 and 2008 of the NuAge study, and available in the NuAge Database.

In the 3C and NuAge studies, all participants signed an informed consent for inclusion. Both studies were conducted in accordance with the Declaration of Helsinki. The 3C study protocol was approved by the Ethical Committee of the University Hospital of Kremlin-Bicêtre. The NuAge Database and Biobank was approved by the Research Ethics Board (REB) of the CIUSSS-de-l’Estrie-CHUS (Quebec, Canada). The present study obtained approval from the REB of the Centre de Santé et de Services sociaux-Institut Universitaire de Gériatrie de Sherbrooke (CSSS-IUGS).

### 2.2. Cognitive Assessment

In 3C, trained psychologists administered a battery of neuropsychological tests at baseline and follow-up examinations, including the Mini-Mental State Examination (MMSE) [17]. The MMSE is a 30-point validated questionnaire, used extensively in research and clinical assessment to screen for dementia and to assess global cognitive function. Participants suspected of dementia on the basis of their cognitive performance were examined by a neurologist [14]. An independent committee of neurologists reviewed potential cases of dementia to reach a consensus based on published criteria.

In NuAge, as mentioned previously, global cognitive function was assessed using the 3MS, the extended version of the MMSE [15]. This neuropsychological test was administered from examinations T1 to T4 by trained research assistants [15]. The MMSE scores can be computed from the 3MS data. Therefore, in order to assess global cognitive function the same way in both cohorts, we used the MMSE scores.

### 2.3. Dietary Assessments

In 3C, individual nutrient intakes were computed from the 24-h dietary recall performed at wave 1 (excluding weekend days) using the BILNUT^®^ software, which converts food intake data into nutrient intake data using French food composition tables [18]. These tables were augmented for fatty acids from the Food Composition and Nutrition tables [19]. As the 24-h recall was open-ended, additional data were also obtained by consulting a French table developed by the INSERM and the University of Montreal [20], the USDA National Nutrient Database, food packaging, and by directly contacting food manufacturers [21,22].

In NuAge, nutrient intakes were computed annually from three non-consecutive 24-h dietary recalls (one face-to-face and two telephone interviews). Analyses of the 24-h dietary recalls have been performed using the CANDAT-Nutrient Calculation System (version 10, Godin, London, ON, Canada) based on the 2007b version of the Canadian Nutrient file (CNF), that was completed by another food composition table of 1200 additional foods that was developed on-site [23]. In order to increase the comparison between the two cohorts, we used only the data from the first 24-h recall administered face-to-face at T1 of NuAge.

### 2.4. Other Variables

In both cohorts, living arrangement was categorized according to two modalities: Living alone vs. living with a relative or an unrelated person. Smoking status was classified as current, ever, or never smoker. For the purpose of this study and to allow comparison with previous studies, education was categorized as 0–6 years, 7–9 years, 10–13 years, and >13 years. Body mass index (BMI) was computed as the weight (kg)/height (m^2^). Additional potential confounders included self-reported history of stroke (yes/no), self-reported diabetes (yes/no), or hypertension (yes/no) at baseline (i.e., wave 1 for 3C, and T1 for NuAge).

### 2.5. Statistical Analyses

**Figure 1:**
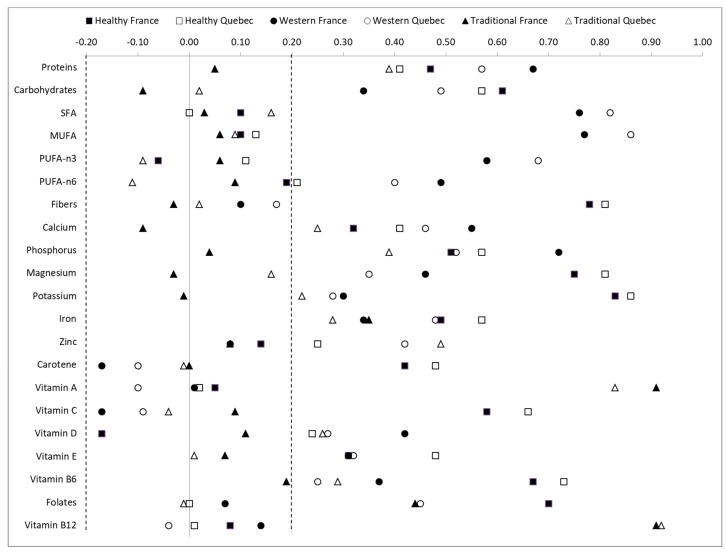
Comparison of the factor loadings from the nutrient patterns obtained in the 3C and NuAge cohorts. SFA: Saturated fatty acids, MUFA: Monounsaturated fatty acids, PUFA: Polyunsaturated fatty acids. Doted lines represent the cut-off values for the interpretability of factor loadings (0.20 in absolute value).

Factor analyses using principal component analysis (FA-PCA) with VARIMAX rotation were previously performed separately in each sample to define nutrient patterns, according to a methodology described previously [12]; the resulting nutrient pattern factor scores were the main explanatory variables in the present study. Briefly, factor scores were obtained from 21 nutrients common in both study samples that are presented in Figure 1. Three factors were identified in each sample according to their eigenvalues, their interpretability, and the percentage of variance explained. The factor scores obtained from FA-PCA were adjusted for total energy intakes using the residual method [24]. The relevant factors identified accounted for very similar total variance in the two study samples (around 50%).

The associations between nutrient pattern factor scores and repeated MMSE scores were estimated using linear mixed models with random effects. As the distribution of the MMSE scores was highly skewed, the square root of the number of errors was calculated as (30 − MMSE)^1/2^ and used as the dependent variable in mixed models [25]. The beta coefficient for each nutrient factor score represents the association between the factor score and the mean cognitive score at baseline, whereas the factor score × time interaction represents the association between the scores and the slope of cognitive change over time. Random effects, including random intercept and random slopes, account for interindividual variability. Three separate models were performed using one nutrient pattern score at a time as an explanatory variable.

We used two models to test potential confounding factors. The first model was adjusted for age, sex, education, and energy, and the second model was additionally adjusted for vascular comorbidities (that were previously reported as risk factors for cognitive decline). Other covariates such as BMI (as a continuous variable), smoking status, and living arrangement were further included in mixed models but as no confounding effect was observed, we did not consider them into the final models (data not shown).

## 3. Results

In 3C, 1712 participants had complete dietary intake data and 1597 of them were evaluated for cognitive function at baseline; 73 were excluded because they had been diagnosed with dementia. Among the remaining participants, 1388 had at least two MMSE scores, including one at baseline, with a mean follow-up of 4.5 years (standard deviation (SD), 1.1). The mean age of the participants was 75.7 years (SD, 4.8) and 62.8% were women. Compared to the baseline cohort, participants excluded from the analytical sample (*n* = 209) were significantly older (mean age = 78.4 vs. 75.7 years, *p* for *t*-test < 0.001), had a lower level of education (29.5% were in the lowest level of education vs. 9.4%, *p* for χ^2^ < 0.001), and a lower global cognitive function (mean MMSE = 26.9 vs. 27.6, *p* for *t*-test < 0.001), but showed no difference in the distribution by sex.

Among the 1596 participants with complete dietary intake data in the NuAge study, 92 were excluded because of implausible reports [26]. In this sample, 1439 participants had at least two 3MS scores, including one at baseline, agreed to be included in secondary analyses, leaving participants with a mean follow-up of 2.9 years (SD, 0.5). The mean age of the participants was 74.3 years (SD, 4.2) and 51.8% were women. Compared to the baseline cohort, participants excluded (*n* = 157) were similar in terms of age, sex, and education, but showed slightly lower values of MMSE scores (mean MMSE = 28.0 vs. 28.3, *p* for *t*-test = 0.02).

Baseline characteristics of both study samples are summarized in Table 1. Compared to 3C participants, NuAge participants were younger and less educated, with fewer women. NuAge participants showed, on average, a slightly higher baseline MMSE score (28.3 vs. 27.6), BMI (27.8 vs. 26.1 kg/m^2^), and energy intake (1928 vs. 1620 kcal/day), included fewer participants with self-reported hypertension (47.4 vs. 51.0%), and more participants living with a relative or an unrelated person (68.5 vs. 45.4%), or with a self-reported history of stroke (3.2 vs. 1.7%). No difference between the two study samples was observed for self-reported diabetes.

The first nutrient patterns identified in both cohorts were qualified as “healthy” (healthy-France and healthy-Quebec, Figure 1), and were characterized by higher intakes in carbohydrates, dietary fiber, magnesium potassium, iron, carotene, and vitamins C, E and B6. Differences between the two cohorts were notable for two specific nutrients. Compared to the healthy-Quebec, the healthy-France pattern was negatively associated with vitamin D intake (factor loadings (FLs) of −0.19 vs. 0.22), and positively associated with folate intake (FLs of 0.69 vs. 0.02). The second nutrient pattern identified in both cohorts was qualified as a Western pattern, characterized by higher intakes of proteins, lipids (saturated, monounsaturated, and *n*-3 and *n*-6 polyunsaturated fatty acids (PUFAs)), calcium, phosphorus, magnesium, vitamin D, and folates, but only for the Western-Quebec pattern (FLs for folates of 0.44 vs. 0.08). The third more traditional nutrient pattern in both cohorts corresponded to diets rich in vitamins A and B12. Higher intake of zinc was observed in the traditional-Quebec pattern (FLs of 0.50 vs. 0.07), whereas higher intake of folates was noted in the traditional-South West of France pattern (FLs of 0.45 vs. −0.01). Average nutrient intake across quartiles of the factor scores are presented in Table 2.

The healthy-France pattern was significantly associated with higher cognitive scores at baseline (i.e., fewer number of errors in the MMSE) in the simplest and the fully-adjusted models (Table 3). For example, in model 2, each 1-point increase of the factor score was associated with −0.053 (95% CI −0.089, −0.016) lower square root errors in the MMSE at baseline. In contrast, the Western-France pattern was significantly associated with lower cognitive scores (Table 3). In model 2, each 1-point increase of the factor score was associated with +0.054 (95% CI 0.006, 0.102) higher square root errors in the MMSE at baseline. No association was observed between the traditional-South West of France pattern and cognitive function. Moreover, there was no association between any nutrient pattern and the slope of cognitive decline.

In NuAge, no association was observed between any of the three nutrient patterns and cognitive function at baseline or the slope of cognitive decline (all *p* values >0.10, Table 2).

## 4. Discussion

This study examined the association of nutrient patterns with cognitive function and decline in two cohorts of older persons living in different environments. In the 3C cohort, the healthy pattern was associated with a slightly higher cognitive performance at baseline, whereas the Western pattern was associated with a slightly lower cognitive performance. We found no relation of nutrient patterns with cognitive function in the NuAge cohort, and no association with cognitive decline was observed in both cohorts. Our study proposes a methodology to harmonize and use dietary and cognitive data from two countries, using nutrient patterns. Another strength of this study is that it is based on dietary data collected by trained dietitians [14,16].

Only one study computed nutrient patterns to estimate their associations with cognitive function in a multiethnic community-based population of 330 older participants residing in Northern Manhattan, NY [12]. This cross-sectional study from the Washington Heights-Inwood Columbia Aging Project (WHICAP) identified an inflammatory nutrient pattern characterized by low intakes of calcium, vitamins D, E, A, B1, B2, B3, B5, B6, folate, *n*-3 PUFAs, and high intake of cholesterol. This pattern was associated with lower cognitive measures of brain health. In our study, healthy nutrient patterns were characterized by lower intakes of vitamin D for the healthy-France pattern, lower intake of vitamin A for both patterns, and lower intake of *n*-3 PUFAs, especially for the healthy-France pattern. Hence, a posteriori nutrient patterns, qualified as “healthy” based on the distribution of specific nutrients considered as “healthy” based on previous knowledge, may not necessarily be optimal for cognitive health. Additionally, the lower intake of folates found in the healthy pattern in NuAge could partially explain why it was not associated with better cognitive function, contrarily to what found in the 3C sample. Indeed, in the 3C study, a higher intake of folates was strongly associated with a lower risk of dementia [27].

Another study based on the WHICAP cohort among 2148 older participants identified a pattern that was significantly associated with lower risk of AD after a mean follow-up of 3.9 years [28]. This pattern was reflecting higher intakes of vitamin E and folates with higher consumption of fish, vegetables, and fruits, and lower intakes of high-fat dairy products and red meat. The healthy-France pattern in our study shares similarities with the dietary pattern identified in the WHICAP (e.g., higher intake of fruits, vegetables, and folates), although it does not include all foods and nutrients that could be protective against cognitive decline (e.g., fish and seafood). This may explain why the positive association between the healthy-France pattern and cognitive performance is small.

The lack of literature on nutrient patterns and cognitive function limits the comparison of our results with other studies. However, some of our findings could be compared to previous studies using dietary patterns. Indeed, nutrient patterns identified in our study were previously described in relation with food intake and overall diet quality [13]. In fact, we identified an opposition between a posteriori healthy and Western nutrient patterns, similar to a posteriori dietary patterns that were previously identified in many epidemiological studies [13]. A similar opposition between two dietary patterns was observed in a cross-sectional analysis based on a sample of middle-aged persons from the Whitehall II study, focusing on cognitive health [29]. These two dietary patterns were also inversely associated with cognitive performance [29]. Indeed, a “whole food” pattern, which was characterized by higher intakes of vegetables and fruits, was reported to be protective for two cognitive domains: Vocabulary and semantic fluency. In contrast, the “processed food” pattern characterized by higher intakes of high-fat and processed foods was associated with higher risk of cognitive impairment for vocabulary and phonemic fluency [29]. Similarly, we observed in the 3C cohort a healthy a priori nutrient pattern associated with better cognitive function at baseline. The plant-based food intakes associated with this healthy a priori nutrient pattern in 3C [13] were similar to those in the whole food dietary pattern from the Whitehall study. This was confirming that a healthy dietary pattern, positively associated with nutrients that were correlated with higher intakes of fruits and vegetables, can benefit cognition.

In our comparative study, a slightly higher baseline cognitive function was associated with a healthy diet, characterized by nutrients and other substances mostly found in plant-based food such as carotene, vitamin E, and dietary fiber, but also folates in 3C. This result is consistent with previous studies suggesting that healthy or prudent diets rich in fruits and vegetables could be protective for cognitive function and risk of dementia [4,7,9,30]. This diet specific to 3C may be protective for cognitive function because of cumulative and synergic effect of nutrients, notably antioxidants, provided by these specific foods. Unexpectedly, in both cohorts, *n*-3 PUFAs, generally recognized as beneficial for cognition [31,32], were not associated with the healthy patterns, but rather with the Western patterns.

Heterogeneity in the results on cognition between the two cohorts may be explained by general health status and different selection criteria at baseline. In NuAge, mean MMSE at baseline was slightly higher than in 3C and the mean decline over time was greater, suggesting that cognitive status baseline in this population was higher than in the 3C population. The proportion of people with a self-reported history of stroke was higher in NuAge, but the proportion of self-reported hypertension was lower. Previous studies on the associations between cognitive performance at midlife have indicated that vascular factors, such as hypertension and diabetes mellitus, could increase the risk of dementia and cognitive decline in later life [33]. Another explanation for the heterogeneity in the results could be related to the diet itself. The adherence to the healthy-France pattern may reflect a better overall nutritional quality for cognitive function. We previously reported that the healthy-France pattern had a stronger positive association with a dietary index estimating the adherence to dietary guidelines, than the healthy-Québec pattern [12]. Finally, follow-up time in NuAge was shorter compared to the 3C cohort, thus reducing the possibility of cognitive decline to occur.

This work has some limitations. First, we were not able to conduct analyses on cognitive domains, unlike other previous studies (e.g., vocabulary or semantic fluency [28]), because different tests were used for these specific domains in the two cohorts and could not be compared. The MMSE is known to show a lack of sensitivity in healthy populations, which translates to ceiling effects, a limitation to detect cognitive changes especially in healthy populations such as those investigated here [34]. However, this test was used in previous studies based on the 3C cohort [35], as well as other cohorts [4,7,11], assessing cognitive changes. Moreover, this test is convenient to use in large-scale epidemiological cohort studies and is internationally validated. This is why it was used for the purpose of data harmonization between the two cohorts, as it was the only common test between them. With regard to potential confounders, apolipoprotein allele e4 (Apoe4) status is a major genetic risk factor for AD [36]. Apoe4 status was available only in the 3C sample at the time of analysis, but was not associated with cognitive function at baseline, or with cognitive decline (data not shown). Finally, serum markers or parameters that were previously reported to be associated with cognitive function, such as HDL-cholesterol [37], other plasma fatty acids [32], or uric acid [38], were not taken into consideration in this comparative study.

## 5. Conclusions

Our results highlight that a nutrient pattern reflecting a balanced diet with a good variety in nutrient intakes, mostly from plant sources, may be associated with higher global cognitive performance, but not with cognitive decline. Comparative studies aimed at assessing external validity of the results in specific populations could be developed in other populations. There is a need for longitudinal cohorts focusing on nutrient patterns with substantial follow-up in order to measure, more accurately, associations between nutrition and cognitive decline in older persons.

## Figures and Tables

**Table 1:** Characteristics of participants of the 3C (*n* = 1388) and the NuAge (*n* = 1439) studies.

	3C		NuAge		*p* *
Age (years), mean (SD)	75.7 (4.8)		74.3 (4.2)		<0.001
Women	872	62.8	746	51.8	<0.001
Education (years)					<0.001
0–6	130	9.4	135	9.4	
7–9	310	22.3	361	25.3	
10–13	383	27.6	503	34.7	
14+	565	40.7	440	30.5	
Living arrangement					<0.001
Alone	757	54.5	454	31.5	
With a relative or non-relative	630	45.4	985	68.5	
BMI (kg/m^2^), mean (SD)	26.1 (4.2)		27.8 (4.4)		<0.001
Energy intake, (kcal/day), mean (SD)	1620 (502)		1928 (642)		<0.001
Smoking					<0.001
Never smoker	862	62.1	742	51.6	
Former-smoker	436	31.4	605	42.0	
Current smoker	88	6.3	92	6.4	
Self-reported hypertension (yes/no) ^†^	708	51.0	682	47.4	0.04
Self-reported diabetes (yes/no) ^†^	130	9.4	155	10.8	0.23
Self-reported history of stroke (yes/no) ^†^	23	1.7	46	3.2	0.001
MMSE at baseline, mean (SD)	27.6 (1.9)		28.3 (1.5)		<0.001

SD: Standard deviation; BMI: Body Mass Index; kcal: Kilocalories; MMSE: Mini-Mental State Examination. Values are represented as *n* and % unless mentioned otherwise. * *t*-test for quantitative variables and χ^2^ for categorical variables. ^†^ self-declared.

**Table 2:** Mean daily nutrient intake per quartile of component scores by nutrient patterns in (**a**) 3C (*n* = 1388) and (**b**) NuAge (*n* = 1439).

(**a**)															
**Mean Intake/Day**	**Healthy-France**					**Western-France**					****	**Traditional-France**			
	**Q1**	**Q2**	**Q3**	**Q4**	*p* *	**Q1**	**Q2**	**Q3**	**Q4**	*p* *	**Q1**	**Q2**	**Q3**	**Q4**	*p* *
Proteins (g)	74.6	70.2	75.1	82.3	<0.001	72.1	71.3	75.6	83.7	<0.001	81.6	72.4	68.3	80.1	<0.001
Carbohydrates (g)	178	181	200	220	<0.001	237	200	182	162	<0.001	238	193	171	179	<0.001
SFA (g)	32.6	25.4	23.9	21.1	<0.001	21.0	24.1	25.8	31.5	<0.001	27.3	23.7	24.6	26.7	<0.001
MUFA (g)	26.9	21.2	19.7	17.9	<0.001	17.0	20.2	21.5	26.5	<0.001	21.4	19.4	20.5	23.9	<0.001
PUFA-n3 (g)	1.9	1.1	1.1	1.0	<0.001	0.7	0.9	1.1	2.3	<0.001	1.2	1.0	1.0	1.8	<0.001
PUFA-n6 (g)	7.2	6.2	6.1	6.4	0.02	5.5	5.7	6.2	8.5	<0.001	6.0	5.7	6.1	8.0	<0.001
Fibers (g)	12	15	18	24	<0.001	23	18	16	14	<0.001	20	17	16	17	<0.001
Calcium (mg)	833	807	883	941	<0.001	736	781	891	1063	<0.001	1096	849	741	783	<0.001
Phosphorus (mg)	1088	1021	1108	1236	<0.001	1049	1031	1116	1266	<0.001	1270	1063	992	1135	<0.001
Magnesium (mg)	222	228	263	315	<0.001	282	251	246	252	<0.001	295	246	234	256	<0.001
Potassium (mg)	2190	2379	2799	3468	<0.001	3121	2692	2583	2495	<0.001	3074	2647	2478	2697	<0.001
Iron (mg)	10.2	9.6	11.0	13.7	<0.001	12.5	10.8	10.4	10.9	<0.001	10.2	9.7	10.3	14.3	<0.001
Zinc (mg)	6.9	7.1	7.7	8.4	0.01	8.2	7.4	7.2	7.4	0.21	6.9	7.3	7.4	8.6	0.01
Carotene (mg)	1489	2188	3231	6891	<0.001	6239	3274	2575	1934	<0.001	4306	3065	3152	3552	0.001
Vitamin A (mg)	700	520	667	877	0.44	1097	507	424	734	0.01	277	258	326	1885	<0.001
Vitamin B6 (µg)	1.2	1.3	1.4	1.8	<0.001	1.7	1.4	1.3	1.4	<0.001	1.5	1.4	1.3	1.6	<0.001
Folates (mg)	199	229	285	389	<0.001	356	268	243	241	<0.001	242	244	265	358	<0.001
Vitamin B12 (µg)	5.9	5.2	5.2	6.4	0.4	6.3	4.7	4.7	7.0	0.01	2.7	2.9	3.6	13.3	<0.001
Vitamin C (µg)	42.7	67.4	86.3	133.9	<0.001	123.0	80.8	70.9	59.3	<0.001	83.2	78.9	82.5	90.2	0.09
Vitamin D (µg)	3.2	1.5	1.3	1.2	<0.001	1.1	1.2	1.4	3.4	<0.001	1.0	1.2	1.6	3.2	<0.001
Vitamin E (µg)	5.8	5.8	6.5	7.7	<0.001	6.5	6.0	6.3	7.2	<0.001	6.3	6.2	6.0	7.5	<0.001
Alcohol (g)	15.1	11.6	11.8	12.7	0.02	16.2	13.6	10.5	10.8	0.006	11.1	11.3	13.6	15.1	0.001
Energy (kcal)	1788	1614	1679	1757	<0.001	1789	1680	1638	1728	0.01	1907	1630	1561	1736	<0.001
(**b**)															
**Mean Intake/day**	**Healthy-Quebec**					**Western-Quebec**					****	**Traditional-Quebec**			
	**Q1**	**Q2**	**Q3**	**Q4**	*p* *	**Q1**	**Q2**	**Q3**	**Q4**	*p* *	**Q1**	**Q2**	**Q3**	**Q4**	*p* *
Proteins (g)	74.6	74.3	78	86.7	<0.001	83.6	73.3	74	82.7	<0.001	67.7	67.5	78.2	101.8	<0.001
Carbohydrates (g)	228.8	219.5	243.4	275.2	<0.001	288.9	233	223.6	220.1	<0.001	279.5	230.7	225.8	230.5	<0.001
SFA (g)	30.8	23.3	20.9	19.3	<0.001	19.4	20	23	31.8	<0.001	24.4	20.4	23	26.6	<0.001
MFA (g)	32	25.5	24.4	24.4	<0.001	22.4	22.7	26.1	35.2	<0.001	30.8	23.8	24.4	27.3	<0.001
PUFA-n3 (g)	1.6	1.4	1.5	1.8	<0.001	1.2	1.3	1.6	2.2	<0.001	2.3	1.5	1.2	1.2	<0.001
PUFA-n6 (g)	12.6	10.4	10.2	9.9	<0.001	8.3	9	10.9	15.1	<0.001	15.5	10.1	8.8	8.5	<0.001
Fibers (g)	13.7	17.8	22	31.2	<0.001	26.5	21.7	19.3	17.2	<0.001	23.6	20.3	19.8	21.1	<0.001
Calcium (mg)	761.3	737.5	793.9	984.3	<0.001	853.8	749	771.9	903.6	<0.001	718.9	695.6	820	1059.6	<0.001
Phosphorus (mg)	1157	1158.3	1306.9	1588.4	<0.001	1400.4	1213.3	1238.1	1359.6	<0.001	1148.1	1131	1268.6	1690.1	<0.001
Magnesium (mg)	250.4	285.5	334.2	432.4	<0.001	381.2	320.7	299.3	300.5	<0.001	332.1	297.7	314.7	361.4	<0.001
Potassium (mg)	2477.6	2884.7	3358.4	4249.2	<0.001	4003.1	3203.4	2943.6	2801.9	<0.001	3206.8	2940	3151.6	3710.3	<0.001
Iron (mg)	12.6	12.2	14	16.6	<0.001	15.5	13.2	12.9	13.8	<0.001	14.2	12.6	13	15.8	0.02
Zinc (mg)	10.8	9.9	10.4	11.5	0.03	11.1	9.8	9.7	12	<0.001	8.7	8.6	10.2	15.2	<0.001
Carotene (mg)	2753	4555.9	7508	14043	<0.001	12390	7711.3	4321.6	4311.7	<0.001	8883.1	6594.4	6459.6	7003.8	<0.001
Vitamin A (mg)	1202.3	893.7	854.8	1162.8	<0.001	1692.6	836.6	757.8	802.2	<0.001	762.9	734.1	875.2	1787.6	<0.001
Vitamin B6 (µg)	1.4	1.6	1.9	2.3	0.09	2.2	1.7	1.6	1.5	<0.001	1.7	1.6	1.7	2.1	<0.001
Folates (mg)	111.8	79.8	81	73.2	<0.001	73.9	72.2	86.6	113.8	<0.001	112	80	76	76	0.01
Vitamin B12 (µg)	5.8	4.4	4.4	4.9	<0.001	7.6	3.6	3.8	4.5	<0.001	2.3	2.9	3.7	10.5	<0.001
Vitamin C (µg)	64.8	94.9	128.6	206.2	<0.001	192.3	124.2	98.3	78	<0.001	150.8	118.2	107.6	116.0	<0.001
Vitamin D (µg)	5.1	4.5	5.2	6.8	<0.001	5.3	4.6	5.2	6.5	<0.001	3.9	4.1	4.9	8.8	<0.001
Vitamin E (µg)	4.7	5.1	5.6	7.9	<0.001	6.3	5.5	5.3	6.2	<0.001	7.3	5.4	5.2	5.5	<0.001
Alcohol (g)	6.8	6.6	5.4	5.5	0.39	10.1	5.9	4.2	3.8	<0.001	6.7	5.6	5.5	6.5	0.48
Energy (kcal)	2028.5	1816.1	1867.3	1998.7	<0.001	2057.1	1779.1	1815.3	2055.7	<0.001	2115	1762	1796	2008	<0.001

Q: Quartile, SFA: Saturated Fatty Acids, MUFA: Monounsaturated Fatty Acids, PUFA: Polyunsaturated Fatty Acids; * *p*-value for ANOVA.

**Table 3:** Associations between nutrient factor scores and baseline cognition, and slope of cognitive change (square root of the number of errors in the MMSE) in mixed models in 3C and NuAge studies.

	Model 1 ^a^		*p*	Model 2 ^b^		*p*
	β	95% CI		β	95% CI	
**3C Study**						
**Healthy-France**						
Factor score on baseline cognition	−0.051	[−0.088;−0.013]	0.007	−0.053	[−0.089;−0.016]	0.005
Factor score * time	0.004	[−0.005;0.014]	0.44	0.004	[−0.005;0.014]	0.39
**Western-France**						
Factor score on baseline cognition	0.059	[0.011;0.107]	0.01	0.054	[0.006;0.102]	0.02
Factor score * time	−0.007	[−0.019;0.005]	0.27	−0.008	[−0.021;0.003]	0.18
**Traditional–South West France**						
Factor score on baseline cognition	0.002	[−0.029;0.033]	0.89	0.002	[−0.029;0.033]	0.89
Factor score * time	−0.002	[−0.010;0.005]	0.56	−0.002	[−0.010;0.005]	0.54
**NuAge**						
**Healthy-Quebec**						
Factor score on baseline cognition	−0.019	[−0.053;0.014]	0.26	−0.020	[−0.055;0.013]	0.23
Factor score * time	−0.002	[−0.017;0.011]	0.23	−0.003	[−0.018;0.010]	0.62
**Western-Quebec**						
Factor score on baseline cognition	0.025	[−0.028;0.078]	0.36	0.025	[−0.029;0.078]	0.36
Factor score * time	0.006	[−0.016;0.028]	0.60	0.006	[−0.016;0.029]	0.57
**Traditional-Quebec**						
Factor score on baseline cognition	0.019	[−0.010;0.050]	0.19	0.019	[−0.010;0.050]	0.19
Factor score * time	−0.002	[−0.015;0.010]	0.72	−0.002	[−0.015;0.010]	0.75

^a^ Adjusted for age, sex, education, and total energy intake. ^b^ Adjusted for age, sex, education, total energy intake, self-reported diabetes or hypertension, and self-reported history of stroke. CI: Confidence interval. Factor score * time = slope of cognitive change.
